# Prenatal methylmercury exposure disrupts developmental trajectories and induces sex-specific toxicity in pubertal rats

**DOI:** 10.21203/rs.3.rs-10208397/v1

**Published:** 2026-07-16

**Authors:** Jessica L. Bradshaw, E. Nicole Wilson, Steve Mabry, Jennifer J. Gardner, Alexandra Grief, Brianne K. Soulen, Celeste L. Ortega-Rodriguez, Tyler D. Armstrong, Amie K. Lund, Aaron P. Roberts, Rebecca L. Cunningham

**Affiliations:** 1 Department of Pharmaceutical Sciences, University of North Texas Health Science Center, 3500 Camp Bowie Blvd, Fort Worth, TX 76107-2699, USA; 2 Advanced Environmental Research Institute, Department of Biological Sciences, University of North Texas, 1155 Union Circle #310559, Denton, TX 76203-5017, USA.

**Keywords:** Methylmercury, perinatal toxicology, developmental programming, developmental delay, puberty, sex differences, metabolic programming, immune programming, public health

## Abstract

**Background:**

Prenatal exposure to the environmental toxicant methylmercury (MeHg) remains a significant public health concern, particularly during vulnerable developmental windows when toxicant susceptibility and sex-specific biology may shape long-term health outcomes. This study examined how a maternal diet spiked with environmentally relevant MeHg levels, including low (400 ppb) and high (800 ppb) concentrations reflecting federal and state consumption advisory guidelines for contaminated fish, affects fetal and postnatal growth, developmental maturation, and circulating biomarkers of liver function, inflammation, and oxidative stress in male and female offspring.

**Methods:**

Timed-pregnant Sprague-Dawley rats were exposed to diets containing vehicle, 400 ppb MeHg, or 800 ppb MeHg from gestational day (GD) 8 through late gestation (GD20) or parturition (GD22–23). Offspring were evaluated for fetal and placental growth (GD20), postnatal developmental milestones (eye opening and pubertal onset), postnatal growth trajectories, pubertal organ weights, and circulating biomarkers during puberty. Total Hg was quantified using direct thermal decomposition, gold amalgamation, and atomic absorption spectrometry. Plasma liver injury biomarkers and cytokine/chemokine profiles were measured using MILLIPLEZ magnetic bead assays. Circulating oxidized proteins were assessed using an advanced oxidation protein products (AOPP) assay.

**Results:**

Gestational MeHg exposure produced dose-dependent Hg accumulation in maternal, placental, fetal, and pubertal offspring tissues, with distinct sex differences in biodistribution. High dose MeHg exposure reduced placental weight but increased placental efficiency and delayed eye opening and male-but not female-pubertal onset. Postnatal growth effects were sex- and dose-dependent, with high dose MeHg-exposed males weighing less and high dose MeHg-exposed females weighing more during late adolescence. Organ weights and circulating biomarkers of liver function (ARG1, SDH, AST, 5’-NT) and inflammatory cytokines (TNF-α, IL-2, IL-1α, IL-17A, IL-6) in pubertal offspring were differentially altered by sex and MeHg dose, indicating persistent metabolic and immune dysregulation.

**Conclusions:**

Environmentally relevant prenatal MeHg exposure disrupts developmental trajectories and induces long-lasting, sex-specific alterations in growth, hepatic function, and inflammatory signaling. These findings highlight sex as a critical biological variable in developmental toxicology and underscore the need to consider sex-dependent mechanisms when evaluating early-life environmental exposures and their long-term health consequences.

## Plain language summary

Mercury (Hg) is a pollutant that builds up in fish and can harm people who eat fish, especially during pregnancy. Methylmercury (MeHg), which is one of the most toxic forms of Hg, can pass from a pregnant mother to her developing baby. This study looked at how a pregnant mother’s diet of MeHg at low, real-world levels (similar to what people might get from eating contaminated fish) affects the growth and health of her offspring, and whether males and females are affected differently. Female rats were given MeHg during pregnancy, and their offspring were tracked from before birth through puberty. We measured growth, when key developmental milestones occurred, how large certain organs were, and whether blood markers showed stress on the liver or immune system.

We found that even low levels of MeHg stayed in the body for a long time and affected development in several ways. Some early developmental milestones, including eye opening and the start of puberty, were delayed. Growth patterns also changed, and these changes were different for males and females. Blood tests also showed signs that MeHg affected the liver and immune system differently in males and females. Overall, our findings show that prenatal exposure to MeHg at levels similar to those found in the environment can have lasting effects on offspring growth and health, and that males and females may not respond the same way. This highlights the importance of protecting pregnant individuals from MeHg exposure and considering sex differences when studying environmental health risks.

## Background

Mercury (Hg) is a persistent environmental contaminant that bioaccumulates and biomagnifies through food webs, posing significant public health risks (1). Hg in the environment is derived largely from anthropogenic activities such as coal combustion, mining, and industrial processes, whereas natural geologic sources contribute a much smaller fraction to the global Hg burden (2). One of the most toxic organic forms of Hg, methylmercury (MeHg), is produced by microbial methylation of inorganic Hg in aquatic environments where it accumulates in fish and seafood, making diet the primary exposure route in humans (1, 3). Although MeHg accumulation is a global problem, human exposure is not evenly distributed. Higher MeHg burdens occur near contamination sites and in communities where reliance on locally sourced fish intersects with limited food alternatives, amplifying environmental health disparities (4–7). These disparities are especially consequential due to the heightened vulnerability of pregnant individuals and developing offspring to environmental toxicant exposure.

Historical poisoning events such as the Japan Minamata disaster, which was caused by industrial discharge of MeHg into Minamata Bay, revealed the profound neurodevelopmental harm of prenatal exposure and catalyzed international policy action (6). Additional disasters, including the Iraq grain-seed MeHg poisoning, further underscored the severe consequences of MeHg poisoning (8). Regulatory efforts, including the Minamata Convention, aim to reduce global Hg emissions and increase public awareness of exposure risks (2, 9, 10). Despite national and global regulatory measures, MeHg exposure remains a persistent public health concern. Hg continues to cycle through long-range atmospheric and aquatic transport and is continually converted to MeHg in aquatic ecosystems, resulting in ongoing and widespread exposure particularly in communities dependent on locally caught fish (2).

During prenatal development, the placenta serves as a critical interface between maternal and fetal environments and is highly sensitive to environmental toxicants (11, 12). MeHg readily crosses both the placental and blood-brain barriers, allowing maternal exposure to directly affect the developing fetus (13, 14). Consistent with the Developmental Origins of Health and Disease framework, prenatal MeHg exposure has been linked to reduced birth weight, impaired growth, and long-term neurodevelopmental deficits (5, 15–21). The Japan Minamata disaster illustrated the severe consequences of gestational MeHg exposure, with profound neurological impairments in exposed children despite relatively mild maternal symptoms (6, 17). While MeHg-induced neurotoxicity is well established, less is known about broader systemic effects of gestational MeHg exposure, including impacts on placental function and offspring growth trajectories, developmental timing, and early-life biomarkers of health and disease.

MeHg can induce systemic toxicity through oxidative stress, inflammation, and metabolic dysfunction. Its affinity for sulfhydryl groups compromises antioxidant defense systems and promotes reactive oxygen species (ROS) generation, leading to cellular damage (22, 23). Furthermore, MeHg exposure has been associated with alterations in inflammatory signaling pathways and liver function (24, 25), suggesting that developmental exposure may have widespread consequences beyond the nervous system. The liver is especially susceptible to MeHg-induced toxicity due to its central role in detoxification, with studies reporting hepatic oxidative injury, altered enzyme activity, and inflammatory responses following exposure (24, 26–30).

Offspring sex is also an important yet often overlooked biological variable in developmental toxicology. Importantly, sex differences in toxicokinetics, placental function, endocrine signaling, antioxidant capacity, and immune responses may influence susceptibility to MeHg during development (31–34). Both epidemiological and experimental studies suggest that prenatal MeHg exposure differentially affects growth, neurodevelopment, and metabolism in males and females, though effects on neuroendocrine maturation and systemic biomarkers remain underexplored (35–39).

Accordingly, the present study investigated the effects of gestational MeHg exposure on perinatal growth, developmental maturation, and circulating biomarkers of liver function, inflammation, and oxidative stress in male and female offspring. Importantly, the MeHg concentrations used were environmentally relevant: the low dose (400 ppb) and high dose (800 ppb) reflect MeHg levels measured in fish from contaminated United States (U.S.) waterways that have prompted state and federal consumption advisories (40–44). Notably, the U.S. Environmental Protection Agency (EPA) employs a national benchmark of 300 ppb for issuing fish consumption advisories, whereas several states adopt substantially higher MeHg thresholds, including Texas at 700 ppb (45, 46). Thus, the developmental effects observed herein occurred at exposure levels relevant to real-world environmental exposures. By using both low and high dose gestational MeHg exposures, the present study was designed to determine whether outcomes vary by dose and whether male and female offspring respond differently across exposure levels. Overall, this study aims to provide a more comprehensive understanding of the systemic consequences of prenatal MeHg exposure at advisory relevant levels and identify potential sex-specific mechanisms underlying developmental toxicity. We hypothesize that prenatal MeHg exposure disrupts offspring developmental maturation and circulating biomarkers of liver function, inflammatory status, and oxidative stress in a sex-specific manner.

## Methods

### Animals and experimental design

Experiments were performed in accordance with ARRIVE and Organization for the Study of Sex Differences (OSSD) guidelines for animal and sex differences research, respectively (47, 48). All rats were provided ad libitum access to standard chow and water in a temperature- and humidity-controlled environment under 12-h:12-h reverse light-dark cycles (lights off 07:00, lights on 19:00). At the conclusion of the study, rats were anesthetized with 2–3% isoflurane during the wake phase (between 09:00 and 16:00), euthanized via decapitation, and tissues were collected and stored at −80 °C until further analysis. Whole trunk blood was collected into EDTA tubes and was either spotted (40 μl in duplicate) onto Whatman 903 Protein Saver cards (Cytiva, Catalog # 10534320) or processed for plasma isolation by centrifuging at 2000×g for 10 min at 4 °C. Isolated plasma supernatants were stored at −80 °C until assayed.

### Dams

Female timed-pregnant Sprague-Dawley rats (aged 3–4 months; Charles River, Wilmington, MA) arrived at the University of North Texas Health Science Center’s animal facility on gestational day (GD) 5 (term = 22–23 days). Dams were single-housed upon arrival and allowed to habituate to the animal facility for three days prior to MeHg exposure (Fig. 1). Two studies were conducted to examine the impact of gestational MeHg exposure on perinatal outcomes (Study 1) and offspring development through puberty (Study 2). Following assignment to Study 1 or Study 2, dams were randomly assigned to Vehicle, 400 ppb MeHg, or 800 ppb MeHg exposure. For Study 1, dams and fetuses were euthanized during late gestation (GD20; term pregnancy = 22–23 days). For Study 2, dams were euthanized at weaning [postnatal day (PND) 21].

### Offspring

For Study 1, fetal and placental weights as well as symmetrical growth assessments (fetal crown to rump length, abdominal circumference, and crown circumference) were measured at GD20 (49–51). For study 2, dams were allowed to give birth (GD 22–23) and litters were reduced within 24 hours after delivery to 8 pups/litter consisting of an equal ratio of males and females. Offspring biological sex was determined on postnatal day 1 (PND1) via anogenital distance and confirmed by gonadal anatomy development prior to weaning. Neurodevelopmental transitions following birth was assessed by quantifying eye opening status with twice daily observations (52). At weaning (PND 21), offspring were cohoused with a same sex littermate (2/cage), with males and females housed in separate rooms to limit any hormonal impacts on study outcomes. Study 2 offspring were grouped based on prenatal MeHg exposure as follows: Male Vehicle (VM, n = 15), Male 400 ppb MeHg (400M, n = 8), Male 800 ppb (800M, n = 8), Female Vehicle (VF, n = 15), Female 400 ppb MeHg (400F, n = 8), and Female 800 ppb (800F, n = 7). Pubertal onset was assessed twice daily by monitoring reproductive anatomical markers (vaginal opening in females; preputial separation in males) (53). Pubertal offspring were euthanized during late adolescence before transitioning into young adulthood (PND 47–48).

### Prenatal MeHg exposure

Pregnant rats received daily exposure to vehicle, 400 ppb MeHg, or 800 ppb MeHg from GD8 through euthanasia (GD20; Study 1) or parturition (GD22–23; Study 2). Doses were delivered via 1 g berry-flavored treats (Bio-Serv, Cat # F05711–1). MeHg-Cl (Sigma Aldrich, Cat # 215465) was dissolved in 70% ethanol and applied slowly to treats; vehicle treats contained ethanol only. All treats were air dried overnight, and a subset of treats was homogenized with a mortar and pestle for total Hg quantification prior to exposure. Rats in the 400 ppb MeHg group received one loaded treat daily at 09:00, whereas the 800 ppb MeHg group received two 400 ppb treats daily at 09:00 and 16:00.

### Mercury quantification

Rat tissue samples and dried blood spots were analyzed for total Hg using a Nippon MA-3000 with direct thermal decomposition, gold amalgamation, and atomic absorption spectrometry (54). Calibration curves used for quantification were built using mercury chloride concentrations ranging from 1 ppb to 10 ppm. The theoretical method detection limit, 0.007 ng Hg, was calculated with 7 replicate blanks as described in Barst et. al. (55). Quality assurance included blanks (empty boat), duplicate samples, and certified reference materials: MESS-3 [marine sediment, certified value = 91 ± 9 ng/g total Hg dry weight (dw)], TORT-2 (lobster hepatopancreas, certified value = 270 ± 60 ng/g total Hg dw), and DOLT-4 (dogfish liver tissue, certified value = 2580 ± 220 ng/g total Hg dw) (National Research Council of Canada). Blank samples had a mean ± SD of 0.003 ± 0.008 ng Hg (n = 45). Mean ± SD percent difference in duplicate samples was 5.82 ± 5.22% (n = 19). The mean percent recovery for MESS-3, TORT-2, and DOLT was 102.7 ± 3.5% (n = 12), 101.1 ± 3.5% (n = 12), and 96.4 ± 6.8% (n = 6).

### Circulating biomarker analysis: liver toxicity, cytokines, and chemokines

MILLIPLEX^®^ (Sigma Millipore) panels were incorporated to quantify plasma levels of rat liver toxicity biomarkers (Cat # RLIMAG-92K) and rat cytokines/chemokines (Cat # RECYTMAG-65 K). Specifically, the liver panel detected plasma levels of arginase −1 (ARG1), aspartate aminotransferase (AST), glutathione s-transferase alpha (GSTα), sorbitol dehydrogenase (SDH), and 5’-neucleotidase (5’-NT). The cytokine/chemokine panel was customized as previously published (49, 56, 57) to detect levels of interleukin (IL)-1α, IL-1β, IL-2, IL-6, IL-10, IL-12p70, IL-17A, IL-18, tumor necrosis factor alpha (TNFα), eotaxin, monocyte chemoattract protein 1 (MCP-1), and macrophage inflammatory protein 1-alpha (MIP-1α). Plasma samples were diluted 1:25 (liver function panel) or 1:2 (cytokine/chemokine panel) in assay buffer prior to running the assay according to manufacturer’s instructions. Samples were run in duplicate and analytes were measured on a Luminex^®^ 200^™^ instrument using xPONENT^®^ software version 4.3 (Luminex Corporation, Austin, TX). Quality control values for all analytes were within the range provided by the manufacturer.

### Circulating advanced oxidation protein products

Plasma levels of oxidized proteins were measured using the OxiSelect Advanced Oxidative Protein Products (AOPP) Assay Kit (Cell Biolabs, Inc., San Diego, CA; Cat # STA-318), incorporating previous modifications (49, 56, 57). Briefly, diluted (1:2) plasma samples were analyzed in a 96-well plate with and without the reaction initiator to correct for background absorbance. AOPP concentrations (μM) were subsequently determined using a 4-parameter logistic standard curve, with background values subtracted from sample readings.

### Statistical analyses

Statistical analyses were performed in Prism v10.6.1 (GraphPad, San Diego, CA). Outliers were identified using the robust regression and outlier (ROUT) method with a coefficient Q = 1% followed by normality assessment using Shapiro-Wilk. Non-Gaussian data were log-transformed or analyzed with non-parametric tests. One- or Two-Way ANOVA (sex, dose) with Fisher’s LSD post-hoc tests were used for parametric tests, while Mann Whitney and Kruskal Wallis with Dunn’s post-hoc tests were used for non-parametric data. The significance level was set to α = 0.05. We report the mean ± standard error (SEM); F, H, or U value; p value; and effect size (η^2^) for significant results. Results of post-hoc testing are displayed in respective graphs with descriptions of comparisons listed within figure legends.

## Results

### Dose-dependent mercury accumulation during gestation disrupts fetal and placental growth dynamics

Maternal dietary intake of 400 ppb (low dose) or 800 ppb (high dose) MeHg resulted in a 20-fold and 36-fold increase, respectively, in total Hg within the maternal muscle during late pregnancy compared to vehicle-dosed control dams (GD20, **Table S1**). Notably, we also observed elevated total Hg in the maternal liver (15-fold increase) and brain (16.5-fold increase) in low dose-exposed dams compared to vehicle-exposed dams. Additionally, we measured total Hg accumulation within a subset of fetal tissues to determine if low dose MeHg exposure resulted in Hg accumulation within fetal-placental units during gestation. Total Hg was increased 17-fold in placenta isolated from a low dose MeHg-exposed dam compared to placenta isolated from a vehicle-exposed dam (GD20, **Table S1**). Moreover, total Hg was also significantly elevated (20-fold) in the fetal brain during gestation (**Table S1**). These total Hg levels remained elevated at 24 hours post-delivery in offspring exposed to low-dose MeHg during gestation (**Table S1**). Dams were kept with their offspring until dam euthanasia at weaning on postnatal day (PND) 21, and total Hg levels were assessed in maternal muscle and liver tissues. At 21 days following the last MeHg exposure, total Hg levels remained elevated in a step-wise, dose-dependent manner within both muscle and liver tissues isolated from dams exposed to high dose MeHg during pregnancy compared to both low-dose and vehicle-exposed dams (**Table S1**).

We evaluated whether MeHg accumulation in tissues was associated with adverse pregnancy outcomes, including altered maternal and pup biometrics, reduced litter size, and asymmetrical fetal growth patterns. Overall, gestational MeHg dietary intake did not affect maternal weight gain, food intake, or water intake over the GD8-GD20 exposure window (**Table S2**). Moreover, there was no effect of dietary MeHg intake on litter sizes (**Table S2**). Similarly, there was no effect of gestational MeHg exposure on fetal weights at GD20 (Fig. 2A). However, we did observe significantly decreased placenta weights in dams exposed to high dose MeHg compared to placentas isolated from both control- and low dose MeHg-exposed dams (Vehicle: 0.47 ± 0.05 g, 400 ppb: 0.46 ± 0.04 g; 800 ppb: 0.39 ± 0.03 g; F = 11.74, p < 0.0001; η^2^ = 0.35; Fig. 2B). Although smaller, the high dose-exposed placentas were more efficient compared to both vehicle and low dose-exposed placentas (Vehicle: 5.29 ± 0.14, 400 ppb: 5.81 ± 0.15, 800 ppb: 6.44 ± 0.18; F = 7.76, p = 0.001, η^2^ = 0.27; Fig. 2C), allowing for maintenance of fetal weights (Fig. 2A). We next examined whether MeHg exposure altered fetal symmetrical growth during pregnancy (Fig. 2 D–F). Although there was no effect on crown to rump length (Fig. 2D) or abdominal circumference (Fig. 2E), we observed dose-dependent alterations in the crown circumference of fetuses exposed to MeHg (Fig. 2F). Particularly, daily prenatal exposure to low dose MeHg resulted in an elevated fetal crown circumference compared to fetuses derived from vehicle or high dose MeHg-exposed dams (Vehicle: 3.15 ± 0.14 cm, 400 ppb: 3.50 ± 0.01 cm, 800 ppb: 3.25 ± 0.06 cm; H = 5.88, p = 0.04, η^2^ = 0.32).

### Gestational MeHg exposure delays offspring neurodevelopmental milestones

To determine if gestational MeHg exposure resulted in long-lasting effects on offspring neuroendocrine developmental milestones, we monitored offspring eye opening and pubertal onset in a subset of offspring exposed to high dose MeHg in utero (Fig. 3). Eye opening was significantly delayed in offspring exposed to high dose MeHg compared to vehicle-exposed offspring (Vehicle: PND 13.52 ± 0.14, 800 ppb: 14.50 ± 0.23; U = 137.5, p = 0.001, η^2^ = 0.43; Fig. 3A). Offspring sex was not determined during monitoring of eye opening to limit disruptions of postnatal maternal care prior to weaning on PND21. After weaning, pubertal onset was measured by examining vaginal opening and preputial separation in females and males, respectively. We observed a significant delay in pubertal onset in 800 ppb MeHg-exposed males compared to vehicle-exposed males (VM: PND 39.43 ± 0.20, 800M: PND 40.00 ± 0.00; U = 12, p = 0.03, η^2^= 0.48; Fig. 3B). However, we did not observe any delays in pubertal onset in female offspring (VF: PND 33.50 ± 0.38, 800F: PND 34.14 ± 0.51; U = 21, p = 0.46; Fig. 3C).

### Gestational MeHg exposure alters offspring postnatal growth patterns in a sex- and dose-specific manner

Postnatal weights were collected during neonatal (PND1), juvenile (PND21), and late adolescence (PND 47–48) life stages. Neonatal male and female offspring exposed to high dose MeHg weighed significantly less than vehicle-or low-dose exposed offspring (VM: 8.23 ± 0.13 g, 400M: 8.52 ± 0.10 g, 800M: 7.62 ± 0.10 g, VF: 7.88 ± 0.10 g, 400F: 7.97 ± 0.10 g, 800F: 6.88 ± 0.27 g; Sex effect: F = 22.10, p < 0.0001, η^2^ = 0.15; Dose effect: F = 22.11, p < 0.0001, η^2^ = 0.33; Interaction: F = 1.66, p = 0.20; Fig. 4A). Juvenile weights reflected neonatal weight assessments, with high dose MeHg exposure resulting in sustained reductions in male and female offspring weights (VM: 58.47 ± 1.60 g, 400M: 56.63 ± 0.80 g, 800M: 50.69 ± 0.62 g, VF: 54.76 ± 1.71 g, 400F: 55.88 ± 0.74 g, 800F: 47.60 ± 1.48 g; Sex effect: F = 3.85, p = 0.05, η^2^ = 0.04; Dose effect: F = 13.78, p < 0.0001, η^2^ = 0.27; Interaction: F = 0.44, p = 0.65; Fig. 4B). A different pattern emerges during late adolescence (PND47–48), whereby the effect of dose is dependent on sex (Sex effect: F = 331.30, p < 0.0001, η^2^ = 0.71; Dose effect: F = 0.05, p = 0.96; Interaction: F = 4.56, p = 0.01, η^2^ = 0.02; Fig. 4C). Specifically, males exposed to high dose MeHg in utero weighed less than vehicle-exposed males, while females exposed to high dose MeHg in utero weighed more than vehicle-exposed females (VM: 274.40 ± 4.38 g, 400M: 269.4 ± 6.25 g, 800M: 260.70 ± 4.20 g, VF: 192.5 ± 3.58 g, 400F: 199.6 ± 3.60 g, 800F: 205.50 ± 2.69 g). Overall, females weighed less than males at all postnatal timepoints, which is supported by the main effect of sex at all timepoints and post-hoc comparisons between male and female vehicle-exposed groups (Fig. 4A–C).

### Dose- and sex-dependent mercury accumulation with organ-specific effects following gestational MeHg exposure

Total Hg levels remained elevated in a dose-dependent manner in pubertal male and female muscle (Dose effect: η^2^ = 0.87) and liver (Dose effect: η^2^ = 0.82) at approximately seven weeks after gestational MeHg exposure (**Table S3**). In the high dose cohort, total Hg was also increased in pubertal male and female brains (Dose effect: η^2^ = 0.97) and whole blood (Dose effect: η^2^ = 0.95). Significant sex differences were observed: females exhibited higher total Hg in the muscle, brain, and whole blood (Sex effect: η^2^ = 0.01–0.02) while males had higher liver total Hg levels (Sex effect: η^2^ = 0.02).

Given MeHg effects on body weight and organ-specific total Hg burden, we also investigated the impact of gestational MeHg exposure on pubertal male and female organ weights (Fig. 5, **Table S4**). Interactive effects of offspring sex and gestational MeHg exposure were detected for normalized brain (Interaction effect: η^2^ = 0.02; Fig. 5A), liver (Interaction effect: η^2^ = 0.13; Fig. 5B), and heart weights (Interaction effect: η^2^ = 0.15; Fig. 5C). High dose MeHg exposure reduced brain weight and increased liver weight in only pubertal females, whereas increased heart weight occurred only in pubertal males. Visceral adipose weights increased in both males and females (Dose effect: η^2^ = 0.07; Fig. 5D). Baseline sex differences were also evident: females exhibited larger normalized brain (Sex effect: η^2^ = 0.68), heart (Sex effect: η^2^ = 0.01), and kidney weights (Sex effect: η^2^ = 0.21), whereas males had larger livers (Sex effect: η^2^ = 0.05) and perigonadal fat (Sex effect: η^2^ = 0.77). No sex or MeHg effects were identified in pubertal offspring lungs.

### Gestational MeHg exposure alters pubertal offspring circulating markers of liver function in a sex- and dose-dependent manner.

Given the liver’s central role in MeHg detoxification, we assessed circulating markers of hepatic stress and injury in pubertal offspring (Fig. 6). Circulating sorbitol dehydrogenase (SDH) was reduced in low dose MeHg-exposed males compared to vehicle-exposed males, with no MeHg effects on plasma SDH levels observed in pubertal females (VM: 3.03 ± 0.67 ng/ml, 400M: 1.17 ± 0.47 ng/ml, 800M: 1.68 ± 0.55 ng/ml, VF: 2.52 ± 0.54 ng/ml, 400F: 1.63 ± 0.63 ng/ml, 800F: 1.70 ± 0.68 ng/ml; Sex effect: F < 0.001, p = 0.99; Dose effect: F = 3.37, p = 0.04, η^2^ = 0.11; Interaction: F = 0.07, p = 0.93; Fig. 6A). Arginase-1 (ARG1) levels were reduced in pubertal male and female offspring exposed to high dose MeHg compared to vehicle-exposed pubertal offspring (VM: 1.02 ± 0.26 ng/ml, 400M: 0.41 ± 0.14 ng/ml, 800M: 0.44 ± 0.15 ng/ml, VF: 0.69 ± 0.14 ng/ml, 400F: 0.72 ± 0.19 ng/ml, 800F: 0.24 ± 0.08 ng/ml; Sex effect: F = 0.03, p = 0.87; Dose effect: F = 5.36, p = 0.008, η^2^ = 0.17; Interaction: F = 1.10, p = 0.34; Fig. 6B). Aspartate aminotransferase (AST) was elevated in high dose MeHg-exposed males compared to vehicle- and low dose-exposed male pubertal offspring with no effects in female pubertal offspring (VM: 28.43 ± 1.28 ng/ml, 400M: 26.97 ± 1.21 ng/ml, 800M: 32.47 ± 0.51 ng/ml, VF: 25.25 ± 0.70 ng/ml, 400F: 25.95 ± 0.70 ng/ml, 800F: 25.48 ± 0.59 ng/ml; Sex effect: F = 12.18, p = 0.001, η^2^ = 0.16; Dose effect: F = 2.08, p = 0.14; Interaction: F = 1.61, p = 0.21; Fig. 6C). Vehicle-exposed pubertal females expressed higher 5’-nucleotidase (5’-NT) plasma levels compared to vehicle-exposed pubertal males, and gestational MeHg exposure induced divergent effects on 5’-NT levels in males and females (Fig. 6D). Specifically, 5’-NT was reduced in low dose-exposed males but elevated in high dose-exposed females. (VM: 2.42 ± 0.12 ng/ml, 400M: 1.81 ± 0.15 ng/ml, 800M: 2.43 ± 0.0.08 ng/ml, VF: 3.16 ± 0.27 ng/ml, 400F: 2.84 ± 0.28 ng/ml, 800F: 5.37 ± 0.58 ng/ml; Sex effect: F = 48.24, p < 0.0001, η^2^ = 0.35; Dose effect: F = 14.52, p < 0.0001, η^2^ = 0.21; Interaction: F = 8.97, p = 0.0004, η^2^ = 0.13). Circulating levels of glutathione s-transferase alpha (GSTα) were below detection limits, which is consistent with its very short plasma half-life and role as a rapid, acute marker of hepatocellular injury (58).

### Gestational MeHg modifies circulating proinflammatory cytokines in pubertal offspring in a sex- and dose-dependent manner.

Gestational MeHg exposure produced sex-and dose-dependent changes in circulating cytokines. Plasma levels of TNF-α were reduced in MeHg-exposed female pubertal offspring at both MeHg doses, with no MeHg exposure effects observed in male pubertal offspring (Sex effect: η^2^ = 0.07; Dose effect: η^2^ = 0.10; Interaction: η^2^ = 0.08; Fig 7A, **Table S5**). IL-2 levels showed bidirectional changes with MeHg exposure in pubertal males (decreased in low dose-exposed, increased in high dose-exposed) but was reduced at both doses in pubertal females (Sex effect: η^2^ = 0.11; Dose effect: η^2^ = 0.16; Interaction: η^2^ = 0.25; Fig. 7G, **Table S5**). Furthermore, gestational MeHg exposure reduced circulating IL-1α (Dose effect: η^2^ = 0.11; Fig. 7D) and IL-17A (Dose effect: η^2^ = 0.16; Fig. 7E), particularly in high dose MeHg-exposed pubertal females, whereas IL-6 levels were increased (Dose effect: η^2^ = 0.11; Fig. 7B), most notably in low dose-exposed pubertal males (trend, p = 0.07). Baseline sex differences were also observed, with vehicle-exposed pubertal females exhibiting higher plasma levels of TNF-α (Fig. 7A), IL-17A (Fig. 7E), and IL-12p70 (Fig. 7F) compared to vehicle-exposed pubertal males. No effects were observed for IL-1β, IL-10, IL-18, or the IL-6 to IL-10 ratio (Fig. 7, **Table S5**). For chemokines, pubertal females expressed higher levels of Eotaxin (Sex effect: η^2^ = 0.14; **Table S5**) while pubertal males expressed higher levels of MCP-1 (Sex effect: η^2^ = 0.08; **Table S5**), with no effects observed on MIP-1α plasma levels (**Table S5**). Circulating oxidized proteins (AOPP) were not altered by offspring sex or gestational MeHg exposure (**Table S5**).

## Discussion

The present study demonstrates that gestational exposure to environmentally relevant doses of MeHg results in persistent Hg accumulation within maternal, placental, fetal, and postnatal offspring tissues and produces long-term alterations in offspring growth, developmental maturation, organ development, and circulating biomarkers of liver function and inflammatory status. Importantly, many of these effects were sex-dependent, highlighting sex as a critical biological variable in environmental Hg toxicology. Collectively, these findings suggest that prenatal MeHg exposure programs physiological systems beyond the central nervous system and may contribute to lasting alterations in offspring growth and metabolic health.

Consistent with previous reports demonstrating efficient placental transfer of MeHg (11, 13), total Hg accumulated within placental tissue and fetal brains during gestation. Notably, we limited MeHg exposure to the gestational period and did not extend maternal exposure into the lactation period when pups were allowed to breastfeed freely. Even so, previous studies have shown very low levels of MeHg are transferred to infants through breast milk, and MeHg levels in mothers and offspring are much higher during birth compared to the breastfeeding period (59, 60). Nonetheless, total Hg remained elevated in offspring tissues through late adolescence, demonstrating the long-term MeHg bioaccumulation in offspring tissues. Our findings also reveal sex differences in MeHg bioaccumulation across organs. Specifically, female offspring exhibited greater Hg accumulation in muscle, blood, and brain tissues, whereas male offspring accumulated more Hg in the liver. Previous findings in young adult rats demonstrated female rats excrete MeHg in the liver at higher rates but retain MeHg at higher levels within the brain (61, 62). Our data extends these findings into early life exposures, suggesting that sex differences in MeHg biodistribution and accumulation occur during vulnerable developmental windows. Still, further studies are warranted to determine if these sex differences are due to differences in MeHg uptake, retention, or excretion rates at various life stages, which may arise from sex differences in early life growth kinetics, metabolism, and transport mechanisms.

We also demonstrate that gestational MeHg exposure disrupts fetoplacental growth dynamics in a dose-dependent manner. Proper fetal development depends on the ability of the placenta to transport essential nutrients required to maintain fetal growth. High dose gestational MeHg exposure significantly reduced placental weight while increasing placental efficiency necessary to maintain fetal body weights. Similar findings have reported impaired placental growth following exposure to environmental toxicants, as well as compensatory placental mechanisms to maintain fetal growth (11, 63–68). Thus, increased placental efficiency may reflect an adaptive response to toxicant-induced placental stress rather than improved placental function. The observation that low dose exposure increased fetal crown circumference without affecting overall fetal weight suggests subtle alterations in fetal growth trajectories that may not be captured by traditional measures of fetal weights. These findings support growing evidence that developmental toxicants can induce asymmetrical growth patterns before obvious fetal growth restriction becomes apparent (68–70).

During postnatal development, prenatal high dose MeHg exposure delayed both eye opening and male pubertal onset, indicating disruption of developmental processes extending beyond gestation. Previous studies have reported no effects of MeHg exposure on offspring eye opening, but this may be due to overt maternal poisoning (e.g., high mg/kg doses) or compounded exposures masking MeHg-specific effects in these previous studies (30, 71). Thus, our findings are the first to identify this developmental delay in response to an environmentally relevant MeHg dose without requiring compounded environmental stressors. This subtle developmental delay also supports previous findings that MeHg exposure disrupts developmental pathways involved in sensory maturation and nervous system development (15, 72–74). We acknowledge that we did not assess sex differences in eye opening; however, we did observe a very large dose effect on eye opening (η^2^= 0.43), suggesting that MeHg exposure may have delayed both male and female eye opening and requires further investigation for confirmation. The delayed pubertal onset observed exclusively in males indicates sex-specific disruption of neuroendocrine maturation. Pubertal timing is tightly regulated by hypothalamic-pituitary-gonadal axis signaling, which is highly sensitive to toxic environmental exposures (75–77). Previous studies have shown that Hg can interfere with endocrine function, steroidogenesis, and reproductive development (78–80). The absence of effects on female pubertal onset suggests that males may be more susceptible to MeHg-induced perturbations of pubertal development, although sex-specific windows of vulnerability and hormonal regulation likely contribute to these differences and require further investigation. Of note, we only assessed these developmental outcomes in high dose-exposed offspring. Thus, it remains unclear if low dose MeHg exposures at EPA-recommended consumption thresholds similarly affect somatosensory and pubertal developmental outcomes.

One of the most notable findings of this study was the emergence of sex-specific growth trajectories following gestational MeHg exposure. Childhood and adolescent growth are important indicators of health and are shaped by both intrinsic factors, such as endocrine and genetic regulation, and extrinsic influences, including nutrition and environmental exposures (81). Postnatal growth follows a characteristic pattern: rapid weight and length gain occur during infancy, growth slows during childhood, and a pubertal growth spurt then drives a marked increase in height and body mass (82). Our findings suggest that gestational MeHg exposure alters puberty-associated weight gain differentially in male and female offspring in a dose-dependent manner. We found that prenatal high dose MeHg exposure reduced body weights in both sexes during the neonatal and juvenile periods. However, by late adolescence, high dose MeHg-exposed pubertal males continued to weigh less than controls whereas high dose MeHg-exposed females exhibited increased body weight. Overall, MeHg suppressed growth in males at all measured life stages, while MeHg-exposed females exhibit suppressed growth during early-life that rapidly accelerated following pubertal onset. These findings extend previous literature by demonstrating that MeHg-mediated growth effects persist beyond childhood and into adolescence. Sex differences in MeHg-altered growth trajectories may reflect differential genetic imprinting, endocrine signaling, or toxicokinetic handling during postnatal development. Particularly in males, MeHg-induced delayed pubertal onset may have also contributed to decreased body weights during late adolescence.

Organ-specific changes following gestational MeHg exposure further support sex-dependent vulnerability to MeHg-induced disruption of developmental growth. Female offspring exposed to high dose MeHg showed reduced normalized brain weight and increased normalized liver weight, whereas males exhibited increased normalized heart weight, indicating that prenatal MeHg exposure can alter organ development in a sex-specific manner. These findings align with prior evidence that developmental toxicant exposure can produce sexually dimorphic effects on organ growth and later cardiometabolic programming (83, 84). In contrast, visceral adiposity increased in both males and females after high dose gestational MeHg exposure, suggesting MeHg induces metabolic disturbances independent of sex during late adolescence. Human cohorts have reported associations between MeHg levels and elevated visceral adiposity in adulthood (85). Our data provides evidence that MeHg-induced elevations in visceral adiposity may stem from developmental programming, with increases occurring prior to adulthood. Indeed, correlative studies in children aged 6–12 have identified positive associations of prenatal MeHg exposure with waist circumference and risk of developing metabolic syndrome (86); still, the role of biological sex was not investigated in these prior studies. Together, these results indicate that gestational MeHg exposure has persistent effects on both organ-specific growth and body composition, with some outcomes showing clear sex dependence and others reflecting a shared susceptibility across sexes.

Having established persistent, sex-dependent effects of gestational MeHg exposure on offspring growth and body composition, we next evaluated whether these systemic disruptions extended to markers of hepatic function. Because the liver is a primary site of MeHg biotransformation and is highly susceptible to MeHg toxicity (87), we assessed circulating biomarkers of liver injury and metabolic stress as indicators of long-term metabolic dysregulation. Gestational MeHg exposure altered several hepatic markers, including decreased ARG1 and altered SDH, indicating persistent impaired metabolic capacity (88, 89). Elevated AST in high-dose males suggests hepatocellular stress, while increased 5′-NT in high-dose females may reflect sex-specific hepatic adaptation or cholestatic processes and biliary tract injury (88, 90). Collectively, these findings indicate that prenatal MeHg exposure programs liver physiology well beyond the exposure window, with effects detectable into adolescence. Prior studies similarly show that Hg exposure can increase liver enzymes and disrupt oxidative and mitochondrial pathways (35, 91), supporting the interpretation that hepatic dysfunction is a durable consequence of developmental exposure.

Developmental MeHg exposure also altered circulating cytokine profiles. Several cytokines, including TNF-α, IL-1α, IL-2, and IL-17A, were reduced in a sex- and dose-dependent manner, while IL-6 increased, particularly in males. This cytokine expression pattern is consistent with evidence that Hg can dysregulate cytokine signaling and induce complex immunomodulatory responses that depend on dose, developmental timing, and cell context rather than simply activating inflammation (92–94). The suppression of several proinflammatory cytokines observed here may reflect altered immune cell development, reduced immune cell signaling capacity, or compensatory adaptation to chronic toxicant exposure. The pronounced sex differences in cytokine responses are particularly noteworthy. Female offspring exhibited higher baseline concentrations of several inflammatory markers and demonstrated greater cytokine suppression following exposure. These findings align with evidence that males and females differ substantially in cytokine production and immune regulation across development (32, 95–99). Interestingly, despite cytokine alterations, circulating advanced oxidation protein products (AOPP) were unchanged. This suggests that systemic oxidative protein damage may not persist into adolescence despite evidence of altered inflammatory and hepatic signaling. Alternatively, persistent tissue-specific oxidative stress may exist but not be reflected in circulating AOPP concentrations.

## Conclusions

Our findings reveal that gestational MeHg exposure produces persistent and sex-dependent effects on offspring development through late adolescence. Prenatal MeHg exposure altered fetoplacental growth dynamics, delayed developmental maturation, disrupted postnatal growth trajectories and organ development, altered liver-associated biomarkers, and modified circulating cytokine profiles. Importantly, many outcomes differed between males and females, highlighting sex-specific susceptibility to gestational MeHg exposure.

## Limitations and future directions

While this study advances understanding of sex-specific developmental responses to prenatal MeHg exposure, several unexamined areas highlight important directions for future research. To begin, mercury speciation was not assessed in offspring tissues, precluding distinction between MeHg and inorganic Hg metabolites. Secondly, evaluation of oxidative stress was restricted to circulating AOPP, which may not adequately capture tissue-specific redox dynamics. In addition, placental molecular mechanisms that may underlie the observed alterations in growth and efficiency were not examined. Similarly, endocrine and metabolic endpoints that could explain the sex-specific growth trajectories were not evaluated, including potential contributions of estrous cyclicity. Moreover, developmental maturation outcomes and tissue weights were examined only in the high dose MeHg exposure group, leaving it unclear whether similar effects occur with low dose MeHg exposure near advisory thresholds. Furthermore, postnatal MeHg exposure via lactational transfer was not quantified. Finally, because outcomes were assessed only through adolescence, it remains unclear whether these effects persist into adulthood; thus, longitudinal studies will be necessary to distinguish transient adaptations from sustained developmental programming effects.

## Supplementary Information

**Table S1.** Total mercury (Hg) accumulation (ppb) in maternal and perinatal offspring tissues.

**Table S2.** Maternal biometrics and litter size.

**Table S3.** Total mercury (Hg) accumulation (ppb) in male and female pubertal offspring.

**Table S4.** Pubertal male and female offspring tissue weights.

**Table S5.** Circulating cytokines, chemokines, and oxidized protein levels in pubertal male and female offspring.

## Figures and Tables

**Figure 1.** Experimental design and timeline. Pregnant rats arrived on gestational day (GD) 5 and were acclimated to animal facilities before treatment began on GD8. Two cohorts were included in the experimental design. Cohort 1 received one treat/day at 09:00 containing vehicle or 400 ppb MeHg; Cohort 2 received two treats/day (09:00, 16:00; Vehicle or 400 ppb MeHg, 800 ppb total exposure). Treatments continued until euthanasia (GD20, Study 1) or pup delivery (GD22–23; Study 2). Litters were culled to 8 pups (4/sex) on PND1 and were weaned from dams on PND21. Eye opening was monitored daily following birth while pubertal onset was monitored after weaning. Vehicle groups from Cohort 1 and Cohort 2 were combined into one vehicle group/sex for comparisons. Pubertal offspring were grouped by sex and treatment into six groups: Vehicle-exposed males (VM; n = 15), vehicle-exposed females (VF; n = 15), 400 ppb MeHg-exposed males (400M; n = 8), 400 ppb MeHg-exposed females (400F; n = 8), 800 ppb MeHg-exposed males (800M; n = 8), and 800 ppb MeHg-exposed females (800F; n = 7).

**Figure 2.** Maternal dietary MeHg disrupts fetoplacental growth dynamics during pregnancy. Fetuses and placentas (n = 12–21/group) were collected from dams exposed to vehicle, 400 ppb MeHg, or 800 ppb MeHg. Individual fetal weights (A), placental weights (B), and fetal to placental weight ratio (C) represent gross fetal and placental biometrics. Symmetrical growth was evaluated in a cohort of each litter (n = 3–8/group) by quantifying fetal crown to rump length (D), abdominal circumference (E), and crown circumference (F). MeHg at 400 ppb increased crown circumference (F) without affecting other measures, while 800 ppb reduced placental weight (B) and increased placental efficiency (C) without altering fetal weight (A). Data are individual fetuses/placentas (sex undetermined). Bars denote means ± SEM. One-Way ANOVA with Fisher’s LSD post-hoc comparisons (A-D); Kruskal-Wallis test with Dunn’s post-hoc comparisons (E-F); *, p < 0.05 vs Vehicle; &, p < 0.05 vs 400 ppb.

**Figure 3.** High dose gestational MeHg exposure delays offspring neuroendocrine development. Maternal intake of 800 ppb MeHg during pregnancy delayed offspring eye opening (A) and male pubertal onset (B), but did not significantly impact female pubertal onset (C). Bars denote means ± SEM; n = 7–24/group. Mann-Whitney test; *, p < 0.05 vs Vehicle.

**Figure 4.** High dose gestational MeHg exposure alters offspring body weight in a sex and age-dependent manner. Gestational exposure to 800 ppb (high dose) MeHg reduced body weight in both male and female offspring at the neonatal [postnatal day (PND) 1; A] and juvenile (PND21; B) stages. At puberty (PND47–48; C), prior high dose gestational MeHg exposure decreased body weight in males but increased body weight in females. In contrast, gestational exposure to 400 ppb (low dose) MeHg did not affect postnatal weight gain. Bars denote means ± SEM; n = 7–26/group. Two-Way ANOVA (sex, dose) with Fisher’s LSD post-hoc comparisons; *, p < 0.05 vs Male Vehicle; #, p < 0.05 vs Female Vehicle; &, p < 0.05 vs same sex 400 ppb.

**Figure 5.** High dose gestational MeHg exposure alters pubertal offspring tissue weights in a sex-specific manner. Gestational exposure to 800 ppb (high dose) MeHg reduced pubertal (postnatal day 47–48) brain weights (A) and increased liver weights (B) in pubertal females, with no effects in male pubertal offspring. Contrarily, high dose gestational MeHg exposure increased heart weights (C) in pubertal males only, while visceral weights were increased in both male and female pubertal offspring. Organ weights were normalized to body weight. Bars denote means ± SEM; n = 6–15/group. Two-Way ANOVA (sex, dose) with Fisher’s LSD post-hoc comparisons; *, p < 0.05 vs Male Vehicle; #, p < 0.05 vs Female Vehicle.

**Figure 6.** Gestational MeHg exposure alters pubertal offspring circulating markers of liver function in a sex and dose-dependent manner. Prenatal low dose MeHg (400 ppb exposure reduced plasma sorbitol dehydrogenase (SDH, A) in males. Prenatal high dose MeHg (800 ppb) exposure decreased arginase-1 (ARG1, B) in both males and females, while increasing aspartate aminotransferase (AST, C) in only males. 5′-nucleotidase (5′-NT, D) was decreased in 400 ppb-exposed males and increased in 800 ppb-exposed females compared to same sex vehicle-exposed rats. Bars denote means ± SEM; n = 7–15/group. Two-Way ANOVA (sex, dose) with Fisher’s LSD post-hoc comparisons; *, p < 0.05 vs Male Vehicle; #, p < 0.05 vs Female Vehicle; &, p < 0.05 vs same sex 400 ppb.

**Figure 7.** Gestational MeHg modifies pubertal offspring circulating proinflammatory cytokines in a sex and dose-dependent manner. Prenatal MeHg exposure reduces plasma levels of TNF-α (A), IL-1α (D), IL-17A (E), IL-12p70 (F), and IL-2 (G) in female pubertal offspring in a dose-dependent manner. Contrarily, prenatal MeHg exposure increases plasma levels of IL-6 (B), IL-12p70 (F), and IL-2 (G) in male pubertal offspring. There were no effects of MeHg dose on plasma levels of IL-1β (C) or IL-10 (H). Similarly, there was no effect of gestational MeHg exposure on the ratio of IL-6 to IL-10 levels (I). Bars denote means ± SEM; n = 7–15/group. Two-Way ANOVA (sex, dose) with Fisher’s LSD post-hoc comparisons; *, p < 0.05 vs Male Vehicle; #, p < 0.05 vs Female Vehicle; &, p < 0.05 vs same sex 400 ppb. IL, interleukin; TNF-α, tumor necrosis factor alpha.

## Data Availability

The datasets used and/or analyzed during the current study are available from the corresponding author upon reasonable request.

## Highlights

- Prenatal MeHg exposure caused sustained, dose-dependent Hg accumulation in maternal, placental, fetal, and pubertal offspring tissues, demonstrating long-term retention after environmentally relevant exposures.
- Pubertal females accumulated more Hg in brain, muscle, and blood, whereas males accumulated more in liver, revealing underrecognized sex-specific toxicokinetics during development.
- Developmental timing was disrupted, with high dose prenatal MeHg exposure delaying eye opening and male (but not female) pubertal onset, underscoring sex-specific vulnerability during neuroendocrine maturation.
- Growth trajectories diverged by sex, with high dose MeHg-exposed males exhibiting lowered adolescent body weight and females exhibiting increased weight, suggesting sex-specific metabolic programming.
- Circulating markers of liver injury and inflammation were altered in sex- and dose-dependent patterns, highlighting persistent disturbances in hepatic and immune regulation.

## Perspectives and significance

This study broadens the understanding of developmental MeHg toxicity beyond its established neurodevelopmental effects. Our findings indicate that prenatal MeHg exposure influences multiple physiological systems, including growth, liver function, and immune regulation, with many outcomes differing between males and females. These results support increasing recognition that environmental toxicants can exert sex-specific developmental programming effects with long-term consequences for health.

Given that human exposure to MeHg primarily occurs through maternal consumption of contaminated fish and seafood, these findings have important implications for pregnancy and developmental health risk assessment. Future studies should investigate the molecular mechanisms underlying sex-dependent susceptibility, evaluate long-term metabolic and reproductive consequences, and identify biomarkers capable of predicting adverse developmental outcomes following prenatal Hg exposure.
